# Quantitative Biomarkers Derived from a Novel Contrast-Free Ultrasound High-Definition Microvessel Imaging for Distinguishing Thyroid Nodules

**DOI:** 10.3390/cancers15061888

**Published:** 2023-03-21

**Authors:** Melisa Kurti, Soroosh Sabeti, Kathryn A. Robinson, Lorenzo Scalise, Nicholas B. Larson, Mostafa Fatemi, Azra Alizad

**Affiliations:** 1 Department of Physiology and Biomedical Engineering, Mayo Clinic College of Medicine and Science, Rochester, MN 55905, USA; 2 Department of Radiology, Mayo Clinic College of Medicine and Science, Rochester, MN 55905, USA; 3 Department of Industrial Engineering and Mathematical Science, Polytechnic University of Marchedelle Marche, 60131 Ancona, Italy; 4 Department of Quantitative Health Sciences, Mayo Clinic College of Medicine and Science, Rochester, MN 55905, USA

**Keywords:** thyroid cancer, ultrasound microvessel imaging, flow imaging, vessel morphological biomarkers

## Abstract

**Simple Summary:**

Low specificity of ultrasound in detecting thyroid cancer warrants the development of new noninvasive modalities for the optimal characterization of thyroid nodules. Here, we present a new ultrasound-based technique, high-definition microvasculature imaging (HDMI) that provides quantitative measures of tumor microvasculature morphological features as new imaging biomarkers. This technique utilizes vessel enhancement filtering, morphological filtering, and vessel segmentation, which enable extraction of vessel morphological features including tortuosity, vessel density, diameter, Murray’s deviation, microvessel fractal dimension, bifurcation angle, number of branch points, and vessel segments. Without the help of contrast agents, through the utilization of HDMI on patients with suspicious thyroid nodules, we were able to resolve tumor microvessels at size scales of a few hundred microns. We further showed that analysis of tumor vessel morphological parameters could detect thyroid malignancy with high sensitivity and specificity. These findings provide a translational rationale for the clinical implementation of quantitative HDMI for thyroid cancer detection.

**Abstract:**

Low specificity in current ultrasound modalities for thyroid cancer detection necessitates the development of new imaging modalities for optimal characterization of thyroid nodules. Herein, the quantitative biomarkers of a new high-definition microvessel imaging (HDMI) were evaluated for discrimination of benign from malignant thyroid nodules. Without the help of contrast agents, this new ultrasound-based quantitative technique utilizes processing methods including clutter filtering, denoising, vessel enhancement filtering, morphological filtering, and vessel segmentation to resolve tumor microvessels at size scales of a few hundred microns and enables the extraction of vessel morphological features as new tumor biomarkers. We evaluated quantitative HDMI on 92 patients with 92 thyroid nodules identified in ultrasound. A total of 12 biomarkers derived from vessel morphological parameters were associated with pathology results. Using the Wilcoxon rank-sum test, six of the twelve biomarkers were significantly different in distribution between the malignant and benign nodules (all *p* < 0.01). A support vector machine (SVM)-based classification model was trained on these six biomarkers, and the receiver operating characteristic curve (ROC) showed an area under the curve (AUC) of 0.9005 (95% CI: [0.8279,0.9732]) with sensitivity, specificity, and accuracy of 0.7778, 0.9474, and 0.8929, respectively. When additional clinical data, namely TI-RADS, age, and nodule size were added to the features, model performance reached an AUC of 0.9044 (95% CI: [0.8331,0.9757]) with sensitivity, specificity, and accuracy of 0.8750, 0.8235, and 0.8400, respectively. Our findings suggest that tumor vessel morphological features may improve the characterization of thyroid nodules.

## 1. Introduction

The incidence of thyroid cancer, the most prevalent endocrine cancer worldwide, has increased in the last few decades [1,2]. Furthermore, thyroid nodules are commonly found in routine physical examinations or as incidental findings on diagnostic imaging performed for other non-thyroidal indications [3]. Clinical examination via palpation is subjective for the detection of thyroid nodules and depends on the experience of the examining clinician, as well as the size and location of the nodule [4]. Although ultrasonography is the first-line imaging tool in evaluating thyroid nodules, the high sensitivity for identifying nodules but unsatisfactory specificity for cancer detection results in overwhelming benign fine needle aspiration biopsy (FNAB), about 60–80% [5]. While FNAB is a widely used and safe procedure, complications such as discomfort or local pain and self-limited small hematomas may occur [6]. The low specificity of ultrasound features for classifying thyroid nodules leads to unnecessary FNABs; therefore, new imaging modalities with special attention to anarchical angiogenesis observed in malignancy are of paramount importance in the characterization of nodules. The addition of strain elastography [7,8], shear wave elastography [9,10,11,12,13], and multimodality ultrasound combining B-mode ultrasound, elastography, and contrast-enhanced ultrasound (CEUS) [14] relatively increased the sensitivity and specificity of ultrasound. 

Thyroid nodules exhibit different patterns of blood flow and vascular morphology that are useful in separating malignant nodules from benign ones [15]. Furthermore, angiogenic activity and sprouting angiogenesis are crucial to thyroid cancer progression [16]. As a consequence of neovascularization, the hallmark of cancer, the microvessel structures in malignant nodules look quite different from those of benign [17]. Imaging modalities that could image and quantify the morphology of tumor microvessels can facilitate cancer diagnosis. Patterns of vascularity, intra-nodular with absent or insignificant perinodular blood flow shown in color Doppler ultrasound and power Doppler ultrasound (PDUS) suggest malignancy [18,19]. With recent advances in slow blood flow imaging, attempts have begun to non-invasively image a tumor’s microvessel structures. With the help of contrast agents, acoustic angiography enables high-resolution imaging of microvasculature [20]. Recently, superb microvascular imaging (SMI) has reported the added value of its microvessel imaging with [21] and without [22,23] TI-RADS for distinguishing benign and malignant thyroid nodules; however, this method is based on visual inspection and quantification is limited to pixel counting. 

A newly developed contrast-free ultrasound-based modality has been introduced to visualize microvessels at a submillimeter level, about 300 μm in diameter [24], labeling it as high-definition microvessel imaging (HDMI) [25]. A series of morphological filtering and vessel enhancement has complemented the HDMI approach to quantify tumor vessel morphological parameters as quantitative vessel biomarkers [24,26,27]. Quantitative HDMI has been tested for distinguishing malignant breast masses from benign ones with remarkable results [25,28].

The objective of the present study is to evaluate the performance of our proposed two-dimensional (2D) quantitative microvessel imaging in classifying malignant and benign thyroid nodules. In this study, microvessel morphological features of thyroid nodules (vessel diameter, tortuosity, vascular density, number of branch points, number of vessel segments, microvessel fractal dimension, bifurcation angle, and Murray’s deviation) are used as HDMI biomarkers. We hypothesized that the aforementioned quantitative biomarkers obtained by HDMI could enhance the differentiation of malignant and benign thyroid nodules with higher specificity. Additionally, significant vessel biomarkers are used to create a model capable of classifying the thyroid nodules as benign or malignant.

## 2. Materials and Methods

### 2.1. Patient Study

This study was performed in compliance with the Health Insurance Portability and Accountability Act (HIPAA) and under the guidelines and regulations of an approved institutional review board (IRB) protocol (IRB#: 08-008778). A written IRB-approved informed consent with permission for publication was obtained from each patient participant prior to the imaging study. Study participants were prospectively enrolled in this study from March 2015 to May 2017. Our study population comprised patients 18 years of age or older with suspicious thyroid nodule(s), who were referred for FNAB. The study participants were previously assigned Thyroid Imaging Reporting and Data System (TI-RADS) assessments by their radiologists based on clinical ultrasound features. TI-RADS scores above 3 were referred for FNAB as part of their clinical care. In addition, nodules with TI-RADS score 3 that were larger than 2.5 cm and those with TI-RADS score 2 but associated with cervical lymphadenopathy and/or a history of thyroid cancer were referred for FNAB. The HDMI study was conducted prior to FNAB. All nodules with positive or indeterminate results of FNABs underwent surgery. The histological results of FNAB and/or surgical pathology reports were utilized as a reference gold standard for malignancy status. The final diagnosis of all malignant cases, regardless of the size, was based on the surgical pathology results—not FNAB. The diagnosis of all benign cases, regardless of the size, was based on the standard clinical findings and FNAB, as there is no need for the benign cases to undergo surgery. 

### 2.2. Clinical Ultrasound Features

All enrolled patients had clinical thyroid ultrasound imaging examinations. TI-RADS points were given based on various ultrasound features in a thyroid nodule. Among the ultrasound features of thyroid; echogenicity (hyperechoic, hypoechoic, or isoechoic); composition (solid or mixed or spongiform); shape (taller than wide); margin (smooth, ill-defined or irregular); calcifications (macrocalcifications, peripheral or rim microcalcification); and vascularity were considered in awarding TI-RADS points to each nodule. The total points in each nodule determined the TI-RADS score. TI-RADS scores were assigned before the HDMI study.

### 2.3. High-Definition Microvasculature Imaging 

For all participants, HDMI was conducted before FNAB. Ultrasound examination was conducted by one of two sonographers with more than 30 and 18 years of experience. Thyroid nodules were identified using an ultrasound platform equipped with plane wave imaging, Alpinion E-Cube 12R ultrasound scanner (Alpinion Medical System Co., Seoul, Republic of Korea), equipped with an L3-12H linear probe operating at 8.5 MHz. Patients were scanned in the supine position with their neck inclined back and turned to the left or right, depending on the position of the nodule. To reduce the compression effect on altering tissue microvessels, our sonographers were instructed to reduce the preload during the ultrasound examination. To diminish motion artifacts, patients were requested to remain at a standstill and pause their breathing for approximately 3 s during data acquisition. After finding the nodule in B-mode ultrasound, a sequence of high frame rate data was processed, detailed in [24,25]. The acquisitions were acquired in both longitudinal and transverse cross-sections of the thyroid gland. Since out-of-plane motion occurs less in longitudinal cross-sections due to the distal location of the trachea and carotid artery with respect to the thyroid gland, the longitudinal view was selected as a more reliable cross-section for microvascular blood flow images. To ensure repeatability, two acquisitions in each orientation were acquired.

After HDMI acquisitions, image processing, and denoising were performed [24,25]. The nodules were manually segmented using B-mode images obtained from the IQ data reconstruction and images were prepared for quantification of morphological parameters of tumor microvessels [26]. All the methods for HDMI image formation, vessel extractions, denoising, morphological filtering, steps for vessel segmentation, and quantification have been detailed in [24,25,26,27,29,30,31,32].

### 2.4. Microvessel Morphological Parameters

Subsequently, after image reconstruction, the morphological parameters of thyroid nodules were extracted from the HDMI images and were used in this study as imaging biomarkers. Vessel density is one of the best-known vessel parameters that defined as the ratio of the geometric area of vessel segments to the geometric area of the associated region of interest of nodule [26,33]. In addition to vessel density, the number of vessel segments (NV), and the number of branch points (NB) that is defined as a common point connected to three or more vessel segments are also calculated [26,34]. Another important parameter, the diameter of the vessel, defined as two times the minimum distance between the vessel centerline and the vessel border has also been calculated [26]. Moreover, we have found that Murray’s deviation (MD), defined as the deviations from Murray’s law, is an important biomarker that presents a diameter mismatch, the definition and calculation of which have been detailed in [27]. Another important vessel morphological parameter, vessel tortuosity determined by distance metric (DM), which is defined as the ratio between the actual path length of a twisting vessel and the linear distance between the two endpoints of that vessel, was measured in this study [26]. Fractal dimension (mvFD), a unitless, geometrical feature that presents the structural complexity of a vascular network has been included in the analysis to provide additional diagnostic and prognostic information. The definition and calculation of mvFD are detailed in [27]. Furthermore, the vessel density ratio (VDR), defined as the ratio of vessel density of the tumor center to the periphery [27], and spatial vascularity pattern (SVP), calculated by VDR, can present the tumor vascular distribution pattern as being either intratumoral or being peritumoral [27,35].

The proposed morphological operations and quantification steps have been well detailed in our previous papers [26,27].

### 2.5. Fine Needle Aspiration Biopsy

All study patients underwent FNAB within a day after the HDMI test. Under ultrasound guidance, our board-certified endocrinologists or radiologists performed FNAB using a standard sterile technique and a 25-gauge needle to obtain six fine needle aspirates for each nodule. Immediately after FNAB, slides were prepared and sent for cytology. Pathological diagnosis was made by pathologists with more than 15 to 20 years of experience. The histopathological results of FNAB and surgical excision for all thyroid nodules were included for data analysis as a reference gold standard.

### 2.6. Statistical Analysis Methods

All image processing and data analyses were performed by the members of our investigative team who were blinded to the pathology results of thyroid FNAB and or surgical pathology. Quantitative variables were summarized as mean ± standard deviation (SD), while nominal variables were summarized as counts and percentages. Differences in distributions of HDMI biomarkers by thyroid nodule malignancy status were assessed using the nonparametric Wilcoxon rank-sum test. A two-sided *p*-value < 0.05 was considered to be statistically significant. To develop a classification model and to investigate the specificity, sensitivity, and area under the curve (AUC) of the receiver operating characteristics (ROC) curve, a multivariable analysis and classifier training were performed using Classification Learner, MATLAB toolbox. Biomarkers with *p*-values less than 0.05 were used as features to train and evaluate classifiers. Subsequent to generating the ROC curves, optimal cut-off thresholds were determined as points with the minimum distance from the maximum sensitivity and specificity point (top left corner) on the curve. 

Data were randomly partitioned into independent subsets to train the algorithm (70%) and the remaining data (30%) for testing. A support vector machine (SVM) classifier with a Gaussian kernel trained in the space of HDMI biomarkers was found to be the best-performing method for our analysis. Two feature sets were considered for model building: the first was restricted to HDMI biomarkers outlined above, while the second considered the addition of the clinical factors of age, nodule size, and TI-RADS. The corresponding models were, respectively, designated as the HDMI model and HDMI-C model. To prevent overfitting during the tuning process, a five-fold cross-validation procedure was used. All of the processes were implemented in MATLAB R2022a (The Mathworks Inc., Natick, MA, USA).

## 3. Results

### 3.1. Characteristics of the Study Population and Thyroid Nodules

A total of 92 patients were successfully enrolled in this study and examined by HDMI. The histopathological results of FNAB confirmed 55/92 (60%) of thyroid nodules as benign, and 2/92 (2%) of the nodules were confirmed to be benign by surgical pathology, constituting a total of 57/92 (62%) benign nodules. All malignant nodules, 35/92 (38%), were confirmed by surgical pathology. From the entire cohort, 74/92 (80%) patients were female and 18/92 (20%) were male, with an age range of 25 to 86 years (mean age ± standard deviation: 53.5 ± 14.8 years). The nodule size in the largest dimension ranged from 6 to 61 mm with a mean ± standard deviation of 21.06 ± 12.70 mm. The participant demographic information, lesion characteristics, and the distribution of malignant lesion type by the pathology are shown in Table 1. The most common malignant histologic type was papillary thyroid carcinoma, corresponding to 31/35 (88%) nodules.

### 3.2. Visualization and Quantification of Microvessel Biomarkers of Thyroid Nodules

**Figure 1:**
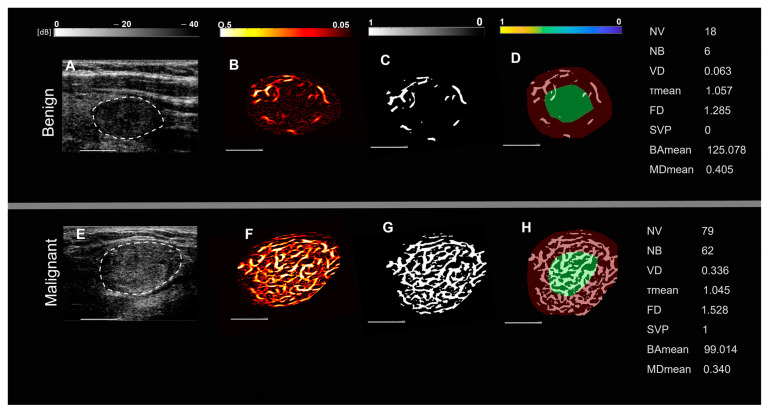
Representative images of large thyroid nodules > 20 mm, benign (top row) and malignant (bottom row). (**A**,**E**) are B-mode ultrasound images (dashed white circles delineate the boundaries of the nodules), (**B**,**F**) are HDMI images showing tumor microvessels, (**C**,**G**) are binary images of microvessels, (**D**,**H**) spatial vascularity pattern (SVP) diagrams (green circles represent the central region and the red ones the peripheral region of the nodule). The correspondent quantitative biomarkers are displayed on the right side of each row. The white line denotes a scale of 1 cm.

**Figure 2:**
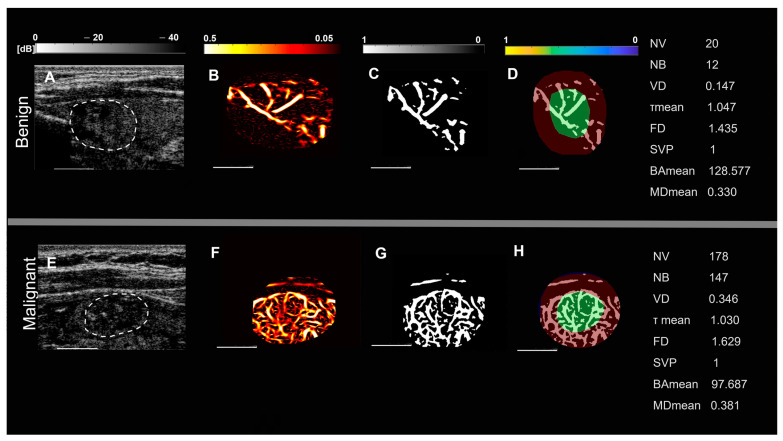
Representative images of small thyroid nodules < 20 mm, benign (top row) and malignant (bottom row). (**A**,**E**) are B-mode ultrasound images (dashed white circles delineate the boundaries of the nodules), (**B**,**F**) HDMI images, showing tumor microvessels, (**C**,**G**) are binary images of microvessels, (**D**,**H**) spatial vascularity pattern (SVP) diagrams, (green circles represent the central region and the red ones the peripheral region of the nodule). The correspondent quantitative biomarkers are displayed on the right side of each row. The white line denotes a scale of 1 cm.

Representative images of benign and malignant thyroid nodules in two groups of patients, based on nodule size and spatial vascularity pattern along with quantified biomarkers are displayed in Figure 1 and Figure 2. These include conventional B-mode ultrasound and HMDI images of larger nodules, with a diameter in the largest dimension, of 24 mm in benign nodules (Figure 1A,B) and 23 mm in malignant nodules (Figure 1E,F), respectively. Figure 2 shows, B-mode ultrasound, and HDMI images of smaller nodules with a diameter in the largest dimension, of 12 mm in benign nodules, Figure 2A,B and 11 mm in malignant nodules, Figure 2E,F. The SVP diagram displays the vascular distribution pattern as perinodular in Figure 1D and intra-nodular in nodules displayed in Figure 1H and Figure 2D,H. The visual inspection shows microvessels with high density along with irregularity in malignant nodules while microvessels in benign are much less dense and more regular. HDMI biomarkers, shown on the right side of Figure 1 and Figure 2 (top) for benign nodules and (bottom) for malignant, demonstrate differentiating values between benign and malignant.

### 3.3. Analysis of HDMI Biomarkers for Differentiation of Thyroid Nodules

**Figure 3:**
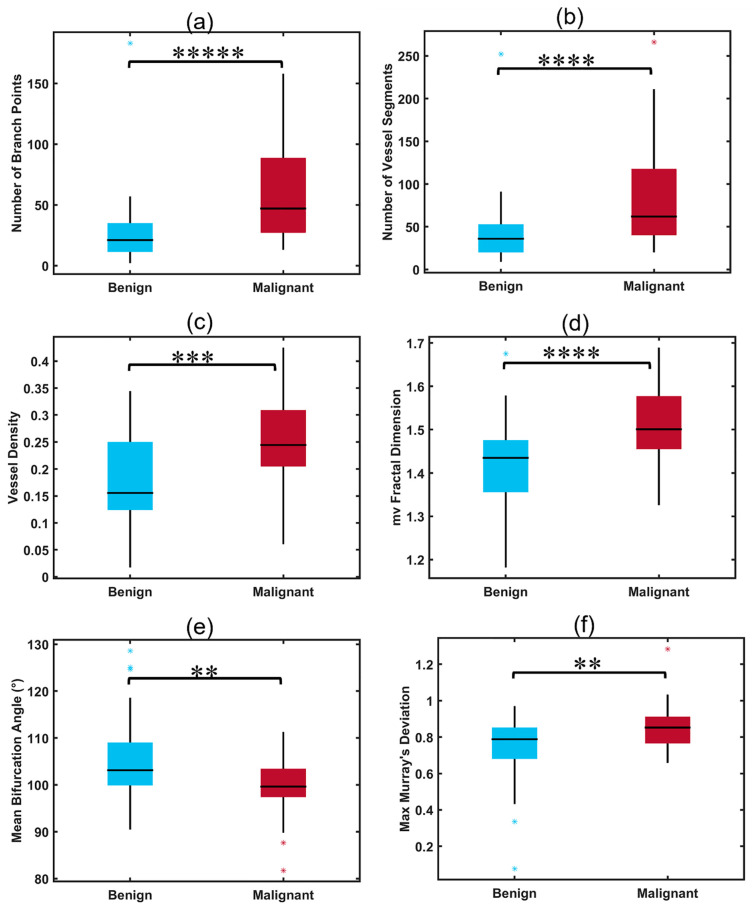
Box plots illustrating distributions of significant HDMI parameters for benign and malignant thyroid nodules. (**a**) Number of branch points (NB), (**b**) number of vessel segments (NV), (**c**) vessel density (VD), (**d**) microvessel fractal dimension (mvFD), (**e**) mean bifurcation angle (BA_mean_), (**f**) maximum Murray’s deviation (MD_max_), ** *p* < 0.01, *** *p* < 0.001, **** *p* < 0.0001, ***** *p* < 0.00001. Benign (*n* = 57), malignant (*n* = 35).

Malignant thyroid nodules had significantly higher values of NV, NB, VD, mvFD, and MD_max_ when compared to benign nodules (all *p* < 0.01, Table 2). The value of BA_mean_ was also a parameter with significantly different distributions between the two groups, showing a decrease in bifurcation angle in malignant nodules when compared to benign ones (*p* = 0.0029). The distributions of HDMI biomarkers with significant differences between malignant and benign nodules are shown in Figure 3 (box plots, a–f).

### 3.4. Differentiating Malignant Nodules from Benign with HDMI Biomarkers, and Combined with Clinical Factors

**Figure 4:**
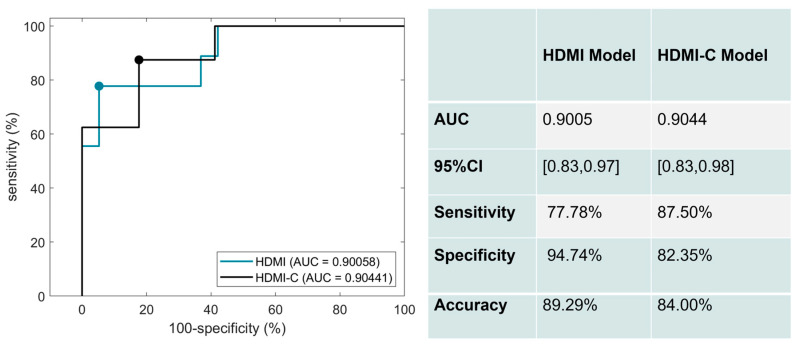
ROC curves generated using the HDMI biomarkers (blue) and HDMI biomarkers combined with clinical data (black).

The corresponding ROC curves for the performance of the HDMI and HDMI-C models on the test set are shown in Figure 4. Six significant HDMI biomarkers were included in the HDMI model. For the HDMI model, the AUC was 0.9005 (95% CI: [0.8279,0.9732]), with a sensitivity of 77.78%, a specificity of 94.74%, and an accuracy of 89.29%. The AUC slightly increased when clinical factors of age, nodule size, and TI-RADS were added to the HDMI model. The corresponding AUC estimate was 0.9044 (95% CI: [0.8331,0.9757]), with a sensitivity of 87.50 %, a specificity of 82.35%, and an accuracy of 84.00%.

## 4. Discussion

The present study evaluated a set of microvessel morphological parameters, extracted from the newly developed US-based quantitative HDMI, as quantitative tumor biomarkers for the differentiation of malignant and benign thyroid nodules. No contrast agent was applied to extract submillimeter microvessels. We identified six HDMI biomarkers, namely vessel density, number of vessel segments, number of branch points, microvessel fractal dimension, bifurcation angle (mean), and Murray’s deviation (max) that demonstrated significantly different distributions between malignant and benign nodules. It is known that the color Doppler flow pattern has very limited value in differentiating benign from malignant thyroid nodules [36]. Efforts have been made to investigate the value of microvessel imaging without the help of contrast agents for thyroid nodule differentiation, either using AngioPLUS Microvascular Imaging [37] or superb microvessel imaging (SMI) [22,38,39]; however, the evaluation is mostly based on visual inspection and the quantification is limited to vessel index and pixel counting. Furthermore, studies on super-resolution imaging combined SMI and CEUS to achieve better results in differentiating benign from malignant nodules [40]; however, the limited quantification along with the inconvenience of injecting contrast agents exists. In support of our observation, Caresio et al. confirmed the correlation between the morphology and distribution of blood vessels and the malignancy, assessing reconstructed vascular architecture from 3D PDUS and CEUS images of thyroid nodules [35]. A study using SMI reported that the addition of TI-RADS scores to other imaging parameters improved the diagnostic accuracy in distinguishing benign from malignant thyroid nodules [21]. In contrast, our study shows that adding TI-RADS scores to the HDMI parameters increased the sensitivity but reduced the specificity while keeping the overall diagnostic performance in terms of AUC essentially the same. Such changes in the sensitivity and specificity of combined HDMI and TI-RADS scores may be attributed to the high sensitivity and low specificity of the sole TI-RADS in thyroid cancer detection.

The present study demonstrates that benign nodules had lower numbers of vessel segments and branch points compared to malignant thyroid nodules. It is known that the rapid growth of the neoplasm is associated with a greater level of vessel sprouting, resulting in increased *NV* and *NB* in malignant tumors [41]. Our findings are also supported by our previous studies in breast cancer detection [25,28] and studies by others [35,42]. In our study vessel density showed statistically significant differences between benign and malignant nodules. Similar findings have been reported by other studies on thyroid nodule differentiation [35] and in renal cell carcinoma [43].

Additionally, we observed higher values of Murray’s deviation in malignant thyroid nodules, with *MD_max_* showing a statistically significant difference between the two groups. The diagnostic value of *MD* has been demonstrated for different diseases [27,44], indicating that the vascular network of diseased and malignant tissue may show a deviation from Murray’s law [45,46,47]. Moreover, our study found lower levels in the mean of the bifurcation angle in malignant thyroid nodules compared with the benign ones. Similar findings have been reported in previous studies on invasive colon carcinomas [48] and breast cancer detection [27,28], showing smaller angles in the vessel network of malignant tissues and with fewer branches in benign, the bifurcation angle among them is wider. Consistent with previous research on oral cancer carcinoma [49], renal cell carcinoma [50], glioblastoma [51], and breast lesions [25], we found that malignant nodules have significantly higher values of *mvFD* than benign and the difference in distribution among the two groups was statistically significant suggesting that the hypervascularity in malignant nodules is associated with complexity, irregularly branched, and distorted microvessel networks. Similar results were obtained from other studies [25].

To avoid selection bias, the patient selection in the current study was not based on age or gender; rather, adult patients with suspicious thyroid nodules who were referred for FNAB were recruited regardless of age and sex. Malignant cases in the present study comprised 77% women and 23% men. This distribution is in agreement with national trends reported in 2022 cancer statistics [52]. The current study also shows a mean age of 42 years old in the group of patients with malignant thyroid nodules, where this number is close to the national average age of 47 years reported for patients with thyroid cancer [53].

The focus of the present study is to validate the performance of the quantitative 2D HDMI for the differentiation of benign and malignant thyroid nodules. In a previous study, using a motorized 3D technique and linear array transducer, the performance of our new three-dimensional (3D) HDMI for differentiation of breast masses was shown in [54]. The future direction may include developing a new 3D system using a 2D matrix transducer suitable for thyroid imaging and performing quantitative 3D HDMI and morphometric analysis of nodule microvessels in three dimensions to improve the diagnostic performance of HDMI. In the future, conducting a large-scale multi-center clinical trial will allow a more thorough investigation of the role of the new microvessel imaging in differentiating thyroid nodules. It should be noted that there is a potential for data degradation due to the motion caused by the thyroid’s proximity to pulsating carotid artery, thus affecting the visualization of microvessels [55]. In future studies, one may employ motion correction algorithms [32] to improve the performance of HDMI in distinguishing benign from cancerous thyroid nodules.

## 5. Conclusions

The low specificity of traditional ultrasound leads to a great number of unnecessary (i.e., benign) biopsies that causes a significant financial and physical burden to the patients. To overcome the present challenging dilemma, we developed and investigated a new contrast-free ultrasound-based quantitative microvasculature imaging technique for better characterization of thyroid nodules. By assessing the morphological features of tumor microvasculature derived from HDMI images, as new biomarkers, we were able to differentiate between malignant and benign thyroid nodules with high specificity. The results of this study indicate the potential of this technique for the optimum characterization of thyroid nodules. In conclusion, quantitative HDMI could offer a way to accurately characterize thyroid nodules and reduce the number of unnecessary thyroid nodules benign biopsies. 

## 6. Patents

US Patent 11,213,278 and EP 3 634 238 B1, JP 7139357 B2.

## Figures and Tables

**Table 1:** Participant demographic characteristics and malignant nodule types.

*Patients* *n= 92*	*Benign* *n= 57 (62)*	*Malignant* *n= 35 (38)*
Gender		
Female ^a^	47 (82)	27 (77)
Male	10 (18)	8 (23)
Age (y) ^b^	55.9 ± 15.2	42.7 ± 13.6
Nodule size ^b^ (mm), largest dimension	21.75 ± 12.50	19.97 ± 13.12
Ultrasound features		
Echogenicity		
Hypoechoic	35 (62)	30 (86)
Isoechoic	19 (33)	5 (14)
Hyperechoic	3 (5)	0 (0)
Composition		
Solid	44 (77)	32 (91)
Mixed and spongiform	13 (23)	3 (9)
Shape		
Taller than wide	10 (18)	9 (26)
Margin		
Ill-defined, irregular margin	24 (42)	23 (66)
Smooth margin	33 (58)	12 (34)
Calcification		
Peripheral or rim microcalcification	3 (5)	5 (14)
Macrocalcification	7 (12)	9 (26)
No calcification	47 (83)	21 (60)
Hypervascularity	38 (67)	22 (63)
TI-RAD Scores		
2	3 (5)	1 (3)
3	8 (14)	0 (0)
4	27 (47)	9 (26)
5	16 (28)	24 (69)
Not reported	3 (5)	1 (3)
Malignant nodule types		35 (38)
Papillary carcinoma		31 (89)
Medullary carcinoma		3 (9)
Anaplastic carcinoma		1 (3)

^a^ Data are numbers of participants. ^b^ Values indicate mean ± standard deviation. Counts are presented with column percentages in parentheses.

**Table 2:** Summary of HDMI biomarker distributions for benign and malignant thyroid nodules and their corresponding *p*-values.

HDMI Biomarkers	Benign (*n* = 57)	Malignant (*n* = 35)	*p*-Value
*NB*	29.00 ± 8.49	42.50 ± 41.72	<0.00001
*NV*	49.00 ± 1.41	65.50 ± 64.35	0.00004
*VD*	0.17 ± 0.08	0.24 ± 0.07	0.00061
*D_max_ (µm)*	624.59 ± 48.48	624.04 ± 9.65	0.98
*VDR*	0.96 ± 0.23	1.09 ± 0.49	0.28
*τ_max_*	1.36 ± 0.004	1.42 ± 0.14	0.44
*τ_mean_*	1.05 ± 0.01	1.06 ± 0.01	0.70
*mvFD*	1.43 ± 0.01	1.46 ± 0.14	0.00005
*BA_mean_*	108.31 ± 96.00	94.41 ± 6.55	0.0029
*BA_max_*	169.12 ± 2.66	149.11 ± 29.37	0.088
*MD_mean_*	0.36 ± 0.02	0.38 ± 0.03	0.28
*MD_max_*	0.81 ± 0.12	0.84 ± 0.13	0.0055

Quantitative variables were summarized as mean ± standard deviation (SD), while nominal variables were summarized as counts and percentages.

## Data Availability

The data that support the findings of this study are available from the corresponding author upon reasonable request. The requested data may include figures that have associated raw data. As the study was conducted on human volunteers, the release of patient data may be restricted by Mayo policy and needs special requests. The request can be sent to Karen A. Hartman, MSN, CHRC|Administrator-Research Compliance|Integrity and Compliance Office|Assistant Professor of Health Care Administration, Mayo Clinic College of Medicine & Science|507-538-5238|Administrative Assistant: 507-266-6286|hartman.karen@mayo.edu Mayo Clinic|200 First Street SW|Rochester, MN 55905|mayoclinic.org.m. We do not have publicly available Accession codes, unique identifiers, or web links.
